# A Qualitative Approach to Understanding Canadian Healthcare Workers’ Use of Coping Strategies during the COVID-19 Pandemic

**DOI:** 10.3390/ijerph20032551

**Published:** 2023-01-31

**Authors:** Mauda Karram, Andrea M. D’Alessandro-Lowe, Kimberly Ritchie, Andrea Brown, Yuanxin Xue, Mina Pichtikova, Maxwell Altman, Isaac Beech, Heather Millman, Fardous Hosseiny, Sara Rodrigues, Alexandra Heber, Charlene O’Connor, Hugo Schielke, Ann Malain, Ruth A. Lanius, Randi E. McCabe, Margaret C. McKinnon

**Affiliations:** 1 Department of Psychiatry and Behavioural Neurosciences, McMaster University, Hamilton, ON L9C 0E3, Canada; 2 Department of Psychology, Neuroscience and Behaviour, McMaster University, Hamilton, ON L8S 4L6, Canada; 3 Homewood Research Institute, Guelph, ON N1E 6K9, Canada; 4 Trent/Fleming School of Nursing, Trent University, Peterborough, ON K9L 0G2, Canada; 5 Temerty Faculty of Medicine, University of Toronto, Toronto, ON M5S 1A8, Canada; 6 Department of Applied Psychology and Human Development, University of Toronto, Toronto, ON M5S 1V6, Canada; 7 Atlas Institute for Veterans and Families, Ottawa, ON K1Z 7K4, Canada; 8 Institute of Mental Health Research at the Royal, University of Ottawa, Ottawa, ON K1Z 7K4, Canada; 9 Veteran’s Affairs Canada, Ottawa, ON K1H 1A1, Canada; 10 Homewood Health Centre, Guelph, ON N1E 6K9, Canada; 11 Lawson Health Research Institute, University of Western Ontario, London, ON N6C 2R5, Canada; 12 St. Joseph’s Healthcare Hamilton, Hamilton, ON L8N 4A6, Canada

**Keywords:** COVID-19, healthcare workers, coping strategy, stress

## Abstract

Throughout the COVID-19 pandemic, healthcare workers (HCWs) have been exposed to highly stressful situations, including increased workloads and exposure to mortality, thus posing a risk for adverse psychological outcomes, including acute stress, moral injury, and depression or anxiety symptoms. Although several reports have sought to identify the types of coping strategies used by HCWs over the course of the pandemic (e.g., physical activity, religion/spirituality, meditation, and alcohol), it remains unclear which factors may influence HCWs’ choice of these coping strategies. Accordingly, using a qualitative approach, the purpose of the present study was to gain a deeper understanding of the factors influencing HCWs’ choice of coping strategies during the COVID-19 pandemic in Canada. Fifty-one HCWs participated in virtual, semi-structured interviews between February and June 2021. Interview transcripts were analysed through an inductive thematic approach, yielding two primary themes. First, HCWs described an ongoing shift in their approach to coping depending on their mental “bandwidth”, ranging from “quick fix” to more “intentional effort” strategies to engage in proactive strategies to improve mental health. Second, many HCWs identified various barriers to desired coping strategies during the pandemic, including the preponderance of pandemic- and other circumstantial-related barriers. The findings from this study offer a unique understanding of the factors influencing HCWs’ choice of coping strategies under novel and increased stress. This knowledge will be central to developing appropriate forms of support and resources to equip HCWs throughout and after the pandemic period, and in mitigating the potential adverse mental health impacts of this period of prolonged stress and potential trauma.

## 1. Introduction

Throughout the COVID-19 pandemic, healthcare workers (HCWs) have been exposed to stressful pandemic-related situations, including witnessing an increased numbers of deaths, heightened workloads and work hours, working with inadequate supplies of equipment or resources, and fear of contracting and/or transmitting COVID-19 to loved ones [1]. Such experiences pose a serious risk for adverse mental health outcomes, including acute stress, insomnia, moral injury, burnout, post-traumatic stress disorder (PTSD), depression, and anxiety [2,3,4,5,6]. Preliminary research has identified a number of common coping strategies used by HCWs during the pandemic, including physical activity/exercise, religion/spirituality, yoga, meditation, alcohol, and talk therapy [2,6]. Although various types of coping strategies utilized by HCWs during the pandemic have been identified by recent studies, it remains unclear how HCWs selected these coping strategies within the unique context of the COVID-19 pandemic in Canada. Accordingly, the purpose of this study was to gain a deeper understanding of the underlying factors influencing the choice of coping strategies among Canadian HCWs during the COVID-19 pandemic. These findings will be critical in developing evidence-informed resources aimed at addressing factors influencing choices of coping strategies with the hope of improving long-term mental health outcomes for HCWs during the pandemic and beyond.

Given the nature of healthcare occupations, particularly those in hospital settings, many HCWs are familiar with working in stressful situations, including exposure to life-or-death decisions, treating life-threatening injuries, and delivering the necessary care response after natural disasters [7]. The COVID-19 pandemic has posed unique challenges to HCWs, involving years of unpredictability, recurring waves of widespread illness, and increased mortality worldwide [7]. Despite these difficult circumstances, many HCWs have nonetheless worked tirelessly under unique and demanding circumstances involving increased physical and psychological demands resulting in high degrees of stress [8]. The psychological burden of such stress, mediated by factors such as unhealthy coping strategies (e.g., substance use) and sleep disturbance, is a known risk factor for immediate and long-term deleterious mental health consequences [2]. For example, Lai et al. [5] surveyed 1257 HCWs (60.8% nurses, 39.2% physicians) across 34 hospitals in China, revealing symptoms of depression, anxiety, insomnia, and distress, where HCWs involved directly in the care of COVID-19 patients had notably elevated symptoms compared to those who were not in direct care of COVID-19 patients. Similarly, Shechter et al. [2] examined 657 hospital workers in the United States (U.S.) during the COVID-19 pandemic (21.5% attending physicians, 21.5% enrolled house staff, 57% nurses and advanced practice providers) and found 57% of participants screened clinically positive for acute stress, 48% for depression symptoms, 33% for anxiety symptoms, and almost 75% reported moderate (or worse) insomnia symptoms. These findings suggest widespread and significant stress and psychological impacts among HCWs as they have navigated the challenging circumstances associated with the COVID-19 pandemic.

Given the high stress nature of healthcare occupations and the elevated threat of adverse psychological outcomes during the COVID-19 pandemic, HCWs would be expected to utilize coping strategies to mitigate stress. Snyder [9] defined coping as a response to stressful daily and life events aimed at diminishing the corresponding physical, emotional, and psychological burden. Here, coping strategies are beneficial responses to promote long-term well-being in the face of stress, as they effectively reduce the aforementioned burden [9]. Coping strategies vary in the degree of positive outcome they produce depending on influencing factors such as perceived ability to change or the improve the situation [9]. Snyder’s [9] definition refers to a desire to actively cope in response to new, imminent stressors, a desire particularly relevant in the context of the COVID-19 pandemic where HCWs may choose to adjust their coping strategies to adapt to novel stress and changing circumstances in their day-to-day lives. Although Snyder’s [9] definition has since been used as a guide for conceptualizing coping strategies in several recent studies (e.g., [10,11,12]), this is the first study to our knowledge to employ this definition in relation to coping strategies adopted by HCWs during the COVID-19 pandemic, which was marked by novel and unprecedented circumstances and, consequently, stressors.

Notably, ongoing research during the COVID-19 pandemic has revealed various coping strategies that HCWs employ to deal with the stress of the pandemic. For example, Smallwood et al. [6] examined coping strategies from 7846 Australian HCWs (39.4% nurses, 31.1% medical staff, 16.7% allied health, 6.2% administrative staff, 6.7% other) and found the most common coping strategy used was physical exercise, followed by yoga, meditation, interacting with friends and family, alcohol use, and psychological well-being apps. Other reports of HCWs’ use of coping strategies during the pandemic corroborate these findings. Specifically, HCWs worldwide have employed physical exercise, yoga, meditation, interactions with friends and family, alcohol use, humour, religion/spirituality, and therapy as coping strategies during the COVID-19 pandemic [2,6,13]. Critically, however, the literature has also revealed that some HCWs were unable to identify any coping strategies. In particular, Kamberi et al. [14] found that among 410 Albanian HCWs, the majority (64%) were unable to identify or make use of any clear coping strategy to manage increased stress during the pandemic.

Taken together, these findings point towards the urgent need to identify whether Canadian HCWs have coped during the pandemic and, if so, which types of coping strategies they employed, in order to promote further adoption of strategies associated with resilience and enhanced wellbeing. Indeed, understanding how HCWs attempt to individually mitigate adverse experiences is likely to be crucial in identifying the types and duration of different forms of support required to help HCWs cope and achieve positive long-term mental health outcomes throughout and after the pandemic. Despite current knowledge of the types of coping strategies employed by HCWs globally, little is known about the coping strategies used by Canadian HCWs and the factors that influence their choice of specific strategies. Accordingly, the present study aimed to obtain first-hand insight from HCWs to better characterize the use of coping strategies by Canadian HCWs and the factors underlying these behavioural choices during the COVID-19 pandemic.

## 2. Materials and Methods

This study is part of a broader project examining the mental health of Canadian HCWs during the COVID-19 pandemic, where HCWs from across Canada were invited to partake in both a qualitative semi-structured virtual interview and a quantitative mental health survey. This study was approved by the Hamilton Integrated Research Ethics Board (#12667). The analysis included solely qualitative findings. The eligibility criteria included: HCWs (e.g., those who provided direct or indirect patient care, supported patient care, administrators etc.), who worked during the COVID-19 pandemic within Canada, were able to speak and read in English, and were at least 18 years of age. Recruitment consisted of virtual flyer distribution to healthcare organizations and associations, as well as targeted social media ads. Upon receiving electronic informed consent forms from participants, interviews were conducted via Personal Health Information Protection Act (PHIPA)-compliant Zoom video conferencing to ensure the privacy and security of all parties and personal data. An interview guide was developed that included 27 open-ended questions, which was piloted with two HCWs prior to study launch. This study analyses responses prompted by a central question: “Some people use various types of coping strategies when they are experiencing stressful situations. Have you used any particular strategies to help you cope?” Coping strategies mentioned at other instances times during the interview were also included in the analysis. All interviews took place between February and June 2021. This period in Canada was characterized by vaccine approval and initial distributions, increasing SARS-CoV-2 variants of concern, travel restrictions, lockdowns— nationwide and localized, and mask mandates [15]. Interviews were recorded as audio-files and sent to a third-party transcription service who transcribed the audio recording verbatim. Transcripts were manually de-identified by both the coding team and a third-party transcription service.

Data were analysed according to Braun and Clarke [16,17] and their six steps for thematic analysis [18]. The following codebook thematic analysis approached the data inductively, at both the latent and semantic level. In guiding this analysis, sensitizing concepts were derived from a comprehensive review of the literature including existing definitions of coping, mental health difficulties experienced by HCWs prior to and during the pandemic, and the types of coping strategies used by HCWs prior to and during the pandemic. Such knowledge created a context for the data, which gave direction to this analysis. Hence, understanding choices of coping strategies and how they may have changed during the pandemic, as well as why HCW’s chose to either engage in or disengage from coping strategies, formed part of the analytical frame.

To begin data analysis, an immersion into these data allowed for an in-depth familiarization with the information at hand. Two diverse transcripts were selected for the development of the coding framework, using an inductive approach. Two independent coders (K.R. and A.B.) developed a coding tree for the entire interview dataset based on these two representative transcripts using MAXQDA 2020 software [19]. Next, a team of independent coders (M.K., A.M.D., K.R., A.B., M.A., I.B., and H.M.) applied the codes to all transcripts. Three team members (M.K., A.M.D., and K.R.) met to review the initial codes and designate main overarching themes and sub-themes [16,17,18]. Candidate themes were revised and either collapsed, excluded, or separated to create coherent, meaningful, and distinct themes [16,17,18]. The revised themes were considered in relation to the entire dataset to ensure an accurate reflection of the whole dataset and ensure the themes fitted together to tell the overarching story of these data [16,17,18]. Each satisfactory theme was identified and described by the essence of what the theme was about, and what it captured for the accompanying narrative [16,17,18]. Finally, themes were given definite, representative names during the final analysis.

Research rigor was maintained through the five criteria proposed by Lincoln and Guba [20,21]; credibility, dependability, confirmability, transferability, and authenticity. The research team worked closely throughout the duration of the project and maintained prolonged engagement with the subject matter. A detailed audit trail was kept, including details of data collection and analysis, as well as coordination and recruitment. To check the quality of the representation of the data in the codes, coding queries were run in MAXQDA 2020 software [19]. Additionally, the principal investigator maintained reflexivity and transparency by recording field notes, memos, and possible biases in a journal.

## 3. Results

### 3.1. Participants

Fifty-one (N = 51) HCWs participated in this study, the majority of whom were female respiratory therapists residing and working in Ontario. This group represents the majority of participants as, prior to expansion of the study to include all healthcare occupations, the original goal of the larger study was to characterize the experiences specifically of respiratory therapists. Demographic data for the study participants are presented in Table 1.

### 3.2. Qualitative Thematic Analysis

**Scheme 1:**
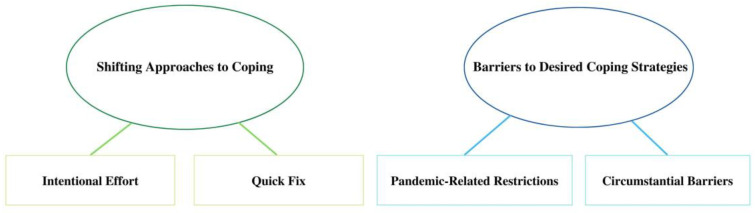
Thematic Map. Note. The final thematic map displays two major themes and their corresponding subthemes. The first theme is Shifting Approaches to Coping, with subthemes Intentional Effort and Quick Fix. The second theme is Barriers to Desired Coping Strategies, with subthemes Pandemic-Related Restrictions and Circumstantial Barriers.

Qualitative thematic analysis revealed two primary themes, each with two subthemes. The themes are not mutually exclusive, but together provided a more holistic understanding of how Canadian HCWs coped during the COVID-19 pandemic. The following Scheme 1 provides an overview of the themes and subthemes observed in the interview data, followed by an in depth analysis and illustrative quotes from participants.

#### 3.2.1. Theme 1: Shifting Approaches to Coping

Despite the described difficulties for HCWs to cope with stress during the pandemic, many HCWs still found ways to use coping strategies. This theme encompasses two main approaches to decision-making which influenced HCWs’ choices of coping strategies. As an overview, the first subtheme, Intentional Effort, refers to HCWs making a purposeful choice to engage in activities that improved their mental health. The associated coping strategies were often described in positive terms (i.e., new enjoyable hobbies or forms of support). The second subtheme, Quick Fix, refers to lower-effort strategies that HCWs engaged in to obtain instant outcomes (e.g., numbing, distraction, relaxation) to cope with stress. These strategies were often described in disparaging terms, although they still offered an instant relief from negative affective states. Importantly, as the pandemic went on, HCWs adjusted their coping strategies according to their needs, presenting fluctuation between the two approaches to decision-making in question. Outlining these shifting approaches to coping, HCWs shared:

“*And to replace the alcohol with things like meditation, I have a sleep routine that I use that [psychologist’s] encouraged me to use so that I sleep well*”

(59RT)

“*Yeah, a few things come to mind. I actually—I—Facebook was a bad one for me, because again, it was like everyone’s opinions on what was going on and I didn’t really want to hear anyone’s opinions, I just wanted the facts. So I actually deleted my account last July after all this stuff happened and I haven’t been back on since, which has been great for me. […] Also, I figured—I came up with new hobbies, because I can’t do [social media], I need to come up with something else. So I started doing more artistic things, like wood burning and carpentry and stuff. And I play piano*”

(20RT)

Here, HCWs chose to terminate their “quick fix” strategies such as alcohol use and social media, and switch to more intentional strategies such as meditation, adequate sleep, and hobbies. The second quote further captures the common fluctuation between approaches to coping as the pandemic went on, where this HCW chose to terminate their social media use due to pandemic-related content and switch to more intentional choices of coping strategies (e.g., wood burning, carpentry, piano, routine social interactions) being described positively. The following sections offer further exploration of what it meant to have a particular approach to coping.

##### Subtheme 1: Intentional Effort 

Many HCWs reported making an intentional effort to invest time and energy to cope with pandemic stress and improve their mental health. Some HCWs described intentionally seeking out coping through personal hobbies, while others leaned towards therapy or routine social interactions. Such strategies were deliberate and planned, demonstrating the intentional effort within the approach to decision making. Many personal hobbies were described positively, including arts, quilting, and gardening as various means to feel better. For example:

“*Gardening too, but now gardening is not all year round. I do have a lot of indoor plants and also we’ve been renovating our house for the last four years so I really much enjoy decorating and changing my decor with the way I feel. So yeah. I think I’m pretty good at coping*”

(47RT)

In describing intentional effort strategies, many HCWs sought out activities that they anticipated would help them feel “happy” or “better” for a prolonged period of time. Some would describe such choices of activities in terms of self-care practices, including yoga, exercise, and meditation. The following HCW described many of these positively perceived activities:

“*Yeah I have started doing like my yoga at home which has been helpful, and I am going to therapy which I started in October, so that’s been helpful for me to do that and I kind of take it as like a self-care kind of day; and like I have been doing little things. I spend a lot of money on like stuff that I enjoy like facials, like skincare kind of stuff just to provide like a little bit of like self-care. […] I’ve seen a lot more like doing a lot more cooking at home and saving more money and, you know, maybe making healthier food choices and working out and having to get creative with workouts and like “I’m going to try some meditation. I’m going to try this actively or watch this video or read this book I’ve been meaning to get to.” Those are great things. Like I’m so happy. A lot of behaviours I think will carry on well past, you know, lockdown and the pandemic*”

(04HCW)

HCWs identified intentional effort strategies as long-term solutions to cope with pandemic stress that were aimed at positively impacting mental health.

##### Subtheme 2: Quick Fix 

By contrast, some HCWs sought lower-effort coping strategies that were more accessible to them. A significant number of HCWs described these strategies as a means to avoid processing their emotions, often due to a lack of bandwidth or capacity to make an intentional effort, and a more urgent need for distraction, relaxation, numbing, and comfort. Some HCWs described an awareness that their quick fix was not a long-term solution, but that it still offered a temporary way to feel comforted or better that was desired in the moment, resulting in continued use. Such coping strategies were often described negatively and were associated with the onset of guilt after being employed. Coping strategies reported as quick fixes included alcohol use, substance use, and binge eating, as illustrated by the following quotes:

“*But it’s hard, you know, some days I just don’t want to do anything and I just want to sit and drink. […] And I guess [alcohol] numbs, right, it kind of takes off that pain that you might be feeling, or that stress, or whatever. It just kind of numbs everything, so yeah*”

(05HCW)

“*I’ve definitely—I don’t know, because I definitely have increased my alcohol definitely and definitely binge eating after a shift. It’s always chips or popcorn and I know that it’s not a long term solution, but I think I just kind of am seeking comfort and that’s going to give me endorphins and I know it’s going to feel good. And then of course that’s—then I feel guilty the next morning and it’s a big old cycle*”

(21RT)

“*Well, I was never a drinker, never one who—well I mean, I very seldom, I mean like once a year would socially have one drink, now I’ve, I could very easily have a couple of drinks a week, or every couple weeks. Again, that could be one cooler or something, which is more than I’ve ever had in my life. And chewing a gummy, CBD, I never did that before. Now, oh yeah, it helps me sleep and some of that. So that’s something I never thought in a million years. But I do that, it’s a coping strategy for me*”

(30HCW)

As evident in the aforementioned example, many HCWs in our study experienced first incidences of increased alcohol and substance use during the pandemic in an attempt to quickly and effectively reduce stress. Moreover, others HCWs engaged in media activities including social media, phone games, and video games. Many HCWs described such experiences:

“*And you don’t have to like turn your brain off to flip through other people’s photos and stuff, so it definitely—like scrolled through Instagram while I was on my break because I couldn’t focus on reading a book. It was just not ideal, but it happened. But, yeah, definitely that was like the bad coping*”

(65RT)

“*I would say probably—yeah, probably more social media again, phone I’m really bad with my phone. It’s more so games than social media though I do like a lot of like puzzles or something. Like I need to be constantly doing something I think to keep my brain busy so, it doesn’t think about other things*”

(52RT)

As HCWs described, activities such as social media required minimal attention and effort, reducing the lingering cognitive load from work, and satisfied more urgent needs (e.g., distraction, avoidance).

#### 3.2.2. Theme 2: Barriers to Desired Coping Strategies

When asked about their use of coping strategies during the pandemic, many HCWs discussed a range of barriers that impeded their usual ways of coping in managing work-related stress. Whereas some barriers were related to pandemic restrictions, others were related to changing circumstances. Oftentimes, barriers were described as out of the individual’s control, affecting their ability to find alternative strategies.

##### Subtheme 1: Pandemic-related restrictions

Government-level policies created prominent barriers to HCWs’ use of coping strategies. All individuals were required to adhere to the policies enacted where they lived (e.g., building closures, limits on social gatherings). Furthermore, there remained prolonged periods of time in which differing degrees of pandemic restrictions were in effect. During these periods, many HCWs were not able to continue or initiate their usual coping strategies, such as physical activity/exercise (i.e., gyms, rock climbing), outings (e.g., restaurants, malls, travel), and social gatherings (e.g., friend’s house, family get-togethers). A few participants shared:

“*And then when you go home, you know, maybe I’ll go out for dinner with some friends. Oh, guess what, we can’t do that. Or maybe I’ll do something as simple as, you know, go bowling and just blow off some steam, have a beer and go bowling with friends. Oh, geez, I can’t do that*”

(05HCW)

“*I sing in like two different choirs, and I’m really missing that because that’s something that I’ve done my whole life and I’ve now learned that it is quite therapeutic for me that whole experience. So, I’m really, really missing that*”

(29HCW)

These HCWs had previous employed coping strategies involving centres in the community (e.g., bowling, concerts, choir), but pandemic restrictions prevented access to them. Similarly, many HCWs spoke about the influence of social restrictions on their ability to utilize desired coping strategies:

“*We weren’t allowed to see our families in some situations. So that was hard on people, because they wouldn’t have the coping mechanisms or the tools that they would normally have*”

(35RT)

“*And I can’t hug my co-worker and we can’t sit in the corner and cry and hug each other, because it’s not allowed. So that kind of support you know. Of course, we talk and try to support each other, but nurses, we’re physical, we need that physical connection and that physical touch, and we can’t have that*”

(05HCW)

Among HCWs who previously relied on contact with family and friends as a prominent coping strategy, this was a great change for many, resulting in negative affective states. The inability to use desired coping strategies took a great toll on many HCWs:

“*So any sort of kind of support mechanisms that we had, anything that … helped to process things and blow off steam, those are all gone. So it’s just kind of in your face, this grief all the time, and there’s no way out to get rid of it, you know*”

(05HCW)

This subtheme addresses the desire of HCWs to engage in coping strategies, which was impeded by various pandemic-related restrictions.

##### Subtheme 2: Circumstantial Barriers

Various circumstances during the pandemic influenced HCWs’ ability to engage in coping strategies to reduce stress. Circumstantial barriers were specific to the HCW’s situation and included communication barriers with family and friends, exhaustion/lack of motivation, and seasonal barriers in terms of weather and the associated available activities. Some HCWs refrained from speaking with friends and family as they were fearful of traumatizing others with their experiences, stories, and challenges, or being portrayed as insensitive to the matter. Other HCWs felt like a burden to their friends and family, or that it would be taxing to speak to friends and family because they would not truly understand their experiences as HCWs. One HCW said:

“*So basically it’s just talking with coworkers and venting it out […] and talk to my family about it, they’re not going to get it because it sounds cold. ‘You want to let someone die?’ ‘Well yeah but that’s in their best interest’. So it sounds cold talking to your family about that so it’s mostly just talking with coworkers. […] Like I think I have a really great family. There is an underlying—they don’t really get it because they’re not there so they can’t, you know, if I had a bad day and I want to come home and talk about it, they can listen and be like “Oh yeah, OK, yeah, uh-huh” but they don’t really get it*”

(10RT)

In addition, HCWs anticipated that differing political opinions about the pandemic, including vaccines and masks, would lead to arguments. These HCWs preferred to avoid these altercations altogether and interacted less with family and friends instead of turning to these relationships for support. One HCW shared:

“*So, of course, [families are] going to talk to a doctor and a nurse about [COVID] and get all the information. And it became very difficult to like talk to family and some friends because that’s all they would want to talk about. And so it was a lot of like we wanted to interact with our families, we want to see our parents, we want to talk to our friends but not like this*”

(02HCW)

Critically, many HCWs were simply too exhausted, burnt out, or unmotivated to engage in any coping strategy at all. At times, participants were unable to fit coping strategies into their schedules due to an increased workload, tending to children at home, and working overtime. Some examples include:

“*And I wish that exercise was a thing, but I used to exercise a lot and kind of—but now it’s just kind of finding the time doesn’t really work out, or when I do have the time I don’t want to*”

(36RT)

“*[…] our kids are small and we would often be like—not necessarily working late, but kind of running from work and straight to like getting them dealt with their schoolwork and dinner and all that kind of stuff. So kind of find a challenge to like where can I fit exercise in? But I find even more so now like I am doing even less [exercise]*”

(08HCW)

Many HCWs were left too burnt out to use any coping strategies, resulting in heightened negative physical and psychological effects. One HCW stated:

“*There was no black and white and then for me it was just that I ended up not coping and then I ended up just not going to work. I don’t know how you cope. I ran out of coping. I felt clearly burnt out. I just felt completely burnt out. I described it as being literally physically and mentally exhausted where I just, I just couldn’t make myself get there and I just couldn’t and right before I went off I actually started to notice and it kind of half scared me*”

(48RT)

Further, many described the winter as a particularly difficult season in coping with pandemic-related stress, as it was too cold to engage in desired outdoor activities, such as going for walks or engaging in socially distanced outdoor gatherings. One HCW explained:

“*But then it got into winter and winter just, with my own personal mental health, it wasn’t good. Winter was terrible. It was a terrible environment at work. It was just a heaviness that winter can be being in a cold place. But it was just…It was just heavy in all regards, lack of sun, lack of being able to social distance outside*”

(12HCW)

The combination of pandemic-related and circumstantial barriers left many HCWs at a standstill, feeling unable to engage in coping strategies to successfully reduce stress.

## 4. Discussion

The purpose of this study was to better characterize Canadian HCWs’ coping strategies during the COVID-19 pandemic and to identify the factors driving selection of and barriers to using particular strategies. To our knowledge, there exists limited literature on Canadian HCWs’ coping strategies during the COVID-19 pandemic, particularly the factors influencing the choices of coping strategies—something which we have elucidated through our rich dataset of HCWs’ recollections of their experiences. With the nationwide impact of the pandemic varying from lockdown periods to new variants of concern, knowledge of coping strategies is of great significance in furthering our understanding of how HCWs’ can best be supported during this time and beyond the pandemic. In this study, most Canadian HCWs interviewed were able to identify strategies they used to combat exposure to stress and potentially, trauma, during the pandemic, with many strategies identified similar to those utilized by non-Canadian HCWs. Here, many of the coping strategies identified by Canadian HCWs were similar to those described in Shechter et al. [2] and Smallwood et al. [6], and included physical exercise, yoga, meditation, interacting with friends and family, and alcohol use. Uniquely, however, the present study identifies potential mechanisms including pandemic-related loss of access and circumstantial barriers, and strategy selection by Canadian HCWs ranging from Quick Fix strategies to strategies involving Intentional Efforts to improve mental health.

In the face of new and difficult circumstances during the pandemic, many Canadian HCWs struggled to find useful means of reducing stress. The current study suggests these struggles were influenced by government restrictions, seasonal barriers, perceived impacts on family and friend relationships, and exhaustion. Few studies have commented on the barriers HCWs experience with regards to choosing and engaging in coping strategies, particularly in relation to the circumstantial barriers identified here. Nonetheless, our study findings align with those of Smallwood et al. [6], who found that a wide selection of Australian HCWs, including nurses, HCWs with children, and HCWs residing in rural areas, found it difficult to maintain social support throughout the pandemic. Notably, the presence of social support is one of the most often identified protective factors against the development of mental health difficulties such as depression, anxiety, and PTSD and trauma-related illnesses in the face of trauma and stress exposure [18,22,23]. Among healthcare workers during the pandemic, barriers such as high at-home responsibilities, high workplace demands, or lockdown restrictions may have significantly impeded access to social support in its various forms [6]. In this study, similar barriers to accessing social support were identified among Canadian HCWs who had difficulty finding time to cope within their busy schedules that included increases in work demands and at-home responsibilities during the pandemic. Moreover, HCWs found it difficult to maintain physical and virtual social connections with friends and family due to what they perceived as high levels of burnout, lack of motivation, lockdown restrictions, and fear of negative impacts on family and friend relationships. Although many of these barriers involved government-imposed restrictions that were out of the control of the individual HCWs, it is important to acknowledge how these restrictions impacted HCWs in terms of coping with novel stressors and to implement resources that can help HCWs navigate through coping and mitigate against the deleterious impact of these barriers.

In the present study, HCWs described frequent fluctuations between Intentional Effort and Quick Fix coping strategies throughout the pandemic. One way that HCWs observed these fluctuations was in terms of their social media use. Many HCWs initially used social media as a coping strategy to “numb out” and distract themselves after a difficult workday, but then moved away from using social media (and engaged in other coping strategies) to avoid the high stress induced from the volume of pandemic-related content on social media. Similarly, Reilly et al. [24] surveyed 888 American HCWs during the COVID-19 pandemic and found that many also decreased their social media usage as a coping strategy; the authors theorized this was a method to avoid adverse mental health outcomes and exposure to tragic events in the media. Along the same lines, Franck et al. [25] originally characterized Facebook and other social media as positive coping behaviours, but found that social media usage was associated with distress among HCWs during the later stages of the COVID-19 pandemic.

This study highlights HCWs’ own perspectives surrounding their use of coping strategies during the COVID-19 pandemic, including what HCWs perceive as positive or negative coping strategies. According to Snyder’s [9] definition, coping strategies are ultimately employed to reduce the physical, emotional, and psychological burden as a result of stressful events. In this study, both negatively and positively perceived coping strategies provided HCWs with their desired outcome of reducing stress, although “quick fix” strategies were often accompanied with negative affective states after-the-fact. As Snyder [9] mentions, coping strategies were indeed adjusted to respond to the imminent threat of novel stress and changing circumstances during the COIVD-19 pandemic. Here, Intentional Effort strategies, such as exercise, meditation, and hobbies were often described in positive terms and outcomes, whereas Quick Fix strategies, such as alcohol and media use, were described in negative terms, although still capable of providing the desired instant and temporary outcomes. In a related study, Reilly et al. [24] found that a quarter of HCW participants described alcohol as a coping strategy and perceived this strategy as somewhat to very effective in managing COVID-19-related anxiety and distress despite what the authors later described as the potential negative outcomes of alcohol use (e.g., increased psychological problems). Argyroula et al. [26] suggest further that COVID-19 was perceived as an uncontrollable and extreme threat to the extent that many people were unsure how to adjust, necessitating adoption of a range of both adaptive and maladaptive coping strategies, where maladaptive strategies were a means to relieve stress quickly, directly, and temporarily. This hypothesis is in line with the present study and may explain, in part, why HCWs continued to engage in strategies such as substance use or social media despite their negative perceptions of these strategies. In addition, HCWs described Quick Fix strategies as easily accessible, avoiding the mental “bandwidth” or motivation necessary to engage in an intentional effort to improve mental health status and perform self-care strategies.

In the present study, many HCWs described their first instances of substance use as occurring during the COVID-19 pandemic, and for HCWs who already engaged in substance use, many described a significant increase in substance use during the pandemic period. It is notable, however, that although some HCWs in our study chose to switch from the Quick Fix of substance use to an Intentional Effort strategy, many continued to rely on substances despite an awareness of the potential for negative long-term impact. This points towards the urgent need for further investigation of HCWs’ substance use during the pandemic. Such work is currently being undertaken in our research group with Patel et al. [27], reporting an association between self-reported PTSD symptoms and alcohol-related problems among HCWs that was mediated by the presence of dissociative symptoms (including the sense that one’s self is not real or the world around oneself is not real). Further research should also monitor long-term substance use among HCWs serving during the pandemic and into its aftermath to aid in identifying not only risk factors for the onset of new or increased substance use, but also prevention and intervention strategies aimed at mitigating potential negative health outcomes among these workers.

Taken together, the results of the present study and a growing body of literature [2,6,20,24] suggest that HCWs are in urgent need of adequate sources of support to assist them with day-to-day coping in view of pandemic-related circumstances and in preventing and mitigating potentially negative long-term mental health outcomes. As leaders with a responsibility to protect HCWs, healthcare organizations must play an integral role in supporting HCWs by promoting evidence-based mental health guidelines. In the face of unforeseen circumstances and closures, appropriate outlets for support should be established to ensure the wellbeing of HCWs in the work environment. Establishing and maintaining balanced daily routines for staff, as well as offering virtual platforms with access to a variety of forms of support, may help combat barriers such as exhaustion, work hours, and seasonal constraints. Positive self-care routines for days off can help create a work-life balance involving adequate nutrition, sleep, exercise, and social connections [28]. Virtual platforms offered twenty-four hours per day may offer HCWs programs and activities (e.g., yoga, meditation, exercise, resiliency and coping training, psychoeducation on stress) that provide coping resources and education, while satisfying any type of work schedule or season.

Significantly, the present study strongly suggests that HCWs have faced numerous challenges to engage in positive coping strategies throughout the pandemic. Addressing these barriers, as much as is realistic and possible, may play an integral role in the development of psychoeducation resources aimed at assisting HCWs during and after the pandemic. Such institutional approaches require adequate funding allocations towards mental health benefits that may provide HCWs with some of the tools they so urgently require. For example, increased benefits for HCWs can help support use of Intentional Effort strategies described in this study to provide positive mental health outcomes (e.g., therapy, physical activity/exercise). Here, the planning of such resources and strategies will need to acknowledge that HCWs fluctuate between an Intentional Effort and a Quick Fix approach to coping, influenced by barriers occurring across the waves of the pandemic. Acknowledging such fluctuations and the decision-making processes that underlie them will be beneficial in understanding HCW perspectives when planning resources and interventions to assist them during and after the pandemic period.

### 4.1. Strengths and Limitations

The results of this study highlight the nuances of coping strategies used by HCWs during the COVID-19 pandemic. Qualitative thematic analysis was utilized to examine HCWs’ first-hand recollections of their experiences during the COVID-19 pandemic. The flexibility of this qualitative approach allowed for an in-depth latent and semantic analysis of transcripts, contributing to expanding our understanding of our findings in relation to barriers and approaches to coping.

Importantly, however, this study is not without limitations. As our sample was composed primarily of a large number of female respiratory therapists residing in Ontario, our results are not generalizable to the entire Canadian HCW population, including those who differ in occupation, sex, gender, ethnic identity, and province or territory of residency. Given the possible influence of geographical location on many of the barriers identified here as impeding coping strategies, it is possible that some of the barriers identified here may not apply to HCWs across Canada. Future research may seek a balanced representation of Canadian HCWs and an understanding of factors influencing the imbalance of demographics of participants. Finally, the present study did not explore organizational or systems-level factors that may significantly influence the coping strategies selected by healthcare workers.

### 4.2. Future Directions

Future work in this area is required to identify differences in psychological (e.g., presence of adverse childhood experiences; severity of psychological symptoms) and demographic (e.g., gender, ethnicity) characteristics that may influence HCWs’ use of various coping strategies, including those that are intended as quick fixes and those that are intentionally oriented. In this regard, the choice of coping strategy may be influenced by the biological, dispositional, personal, and family characteristics an individual brings to a scenario [29,30]. When accounting for factors such as mental health, demographics, previous trauma, family history, and medical history, perceptions of barriers or particular routes of decision-making can be better understood and addressed through resources that address these intersectional identity characteristics. Moreover, given the varying perceptions of HCWs concerning their coping strategies, an objective measurement of their effectiveness may assist in the identification of individual, organisational, and public level strategies aimed at assisting HCWs. Frydenberg [30] considered effective coping strategies to be those that transform given challenges into a solvable problem followed by positive outcomes, whereas maladaptive coping strategies lead to escalations in challenges followed by negative outcomes. While helpful, such characterizations ignore the wider spread realities facing Canadian HCWs including staffing shortages, increased exposure to mortality and widespread concerns about virus transmission to loved ones and others that together may impede access to and use of coping strategies.

## 5. Conclusions

This study indicates that Canadian HCWs faced challenges in making use of usual coping strategies during the pandemic due to various barriers. Nevertheless, HCWs did still engage with coping strategies, shifting between different selection approaches. HCWs fluctuated between Intentional Effort decisions and Quick Fix decisions, influenced by contextual factors. These findings can inform forms of support and resources at both the individual and organizational level to equip HCWs to cope in times of increased stress with the overall aim of improving mental health and wellbeing. Future studies should continue to explore factors involved in choice of coping strategies, as well as identify coping strategies that empirically reduce stress and adverse psychological symptoms among HCWs. Overall, this study underscores the critical need to assist HCWs in coping with occupational stress and the unique psychological and physical burden of the COVID-19 pandemic on them and on their families.

## Figures and Tables

**Table 1:** Participant Demographics.

	Frequency (n = 48)	Percent of Sample
**Occupation**		
Respiratory Therapist	35	68
Nurse	6	13
Other (e.g., personal support worker, medical office assistant, audiologist, hospital physician)	10	19
**Sex**		
Female	42	87
Male	6	13
**Age (years)**		
20–29	9	19
30–39	15	31
40–49	16	33
50+	8	17
**Location**		
British Columbia	5	10
Alberta	8	17
Ontario	32	67
Other	3	6

Note. The table represents the 48 (out of total N = 51) participants who provided demographic data via the quantitative portion of the study.

## Data Availability

The data used in this study come from the McKinnon Trauma and Recovery Research Unit at McMaster University. All interested researchers may apply for access to these data through online application subject to review by the Data Access Committee, ethics approval, and signing of a data sharing agreement. Data are provided only once a data sharing agreement is in place between McMaster University (the custodian of the data) and the researchers’ institution. For more information about data access please contact https://www.thetraumaandrecoverylab.com/contact (accessed 19 December 2022).

## References

[1] Petzold M.B., Plag J., Strohle A. (2020). Dealing with mental stress among health professionals in the context of the COVID-19 pandemic. Neurologist.

[2] Shechter A., Diaz F., Moise N., Anstey D.E., Ye S., Agarwal S., Birk J.L., Brodie D., Cannone D.E., Chang B. (2020). Psychological distress, coping behaviours, and preferences for support among New York healthcare workers during the COVID-19 pandemic. Gen. Hosp. Psychiatry.

[3] Sriharan A., West K.J., Almost J., Hamza A. (2021). COVID-19-related occupational burnout and moral distress among nurses: A rapid scoping review. Nurs. Leadersh..

[4] Khan A.J., Nishimi K., Tripp P., Maven D., Jiha A., Woodward E., Inslicht A., Richards A., Neylan T.C., Maguen S. (2021). Covid-19 related moral injury: Associations with pandemic-related perceived threat and risky and protective behaviours. J. Psychiatr. Res..

[5] Lai J., Ma S., Wang Y., Cai Z., Hu J., Wei N., Wu J., Du H., Chen T., Li R. (2020). Factors associated with mental health outcomes among health care workers exposed to coronavirus disease 2019. JAMA Netw. Open.

[6] Smallwood N., Karimi L., Pascoe A., Bismark M., Putland M., Johnson D., Dharmage S.C., Barson E., Atkin N., Long C. (2021). Coping strategies adopted by Australian frontline health workers to address psychological distress during the COVID-19 pandemic. Gen. Hosp. Psychiatry.

[7] Morganstein J.C., Flynn B.W. (2021). Enhancing psychological sustainment & promoting resilience in healthcare workers during COVID-19 & beyond. J. Occup. Environ. Med..

[8] Babore A., Lombardi L., Viceconti M.L., Pignataro S., Marino V., Crudele M., Candelor C., Bramanti S.N., Trumello C. (2020). Psychological effects of the COVID-2019 pandemic: Perceived stress and coping strategies among healthcare professionals. Psychiatry Res..

[9] Snyder C.R. (1999). Coping: The Psychology of What Works.

[10] Marciano H., Eshel Y., Kimhi S., Adini B. (2022). Hope and fear of threats as predictors of coping with two major adversities, the COVID-19 pandemic and an armed conflict. Int. J. Environ. Res. Public Health.

[11] Pinedo R., Vicario-Molina I., Ortega E.G., Picos A.P. (2021). Factors related to mental health during the COVID-19 lockdown in spain. Front. Psychol..

[12] Prasath P.R., Bhat C.S., Mather P.C., Foreman T., James J.K. (2021). Wellbeing, psychological capital, and coping of university employees during the COVID-19 pandemic. J. Professiorate.

[13] Vancappel A., Jansen E., Ouhmad N., Desmidt T., Etain B., Bergey C., d’Ussel M., Krebs M., Paquet C., Réveillère C. (2021). Psychological impact of exposure to the COVID-19 sanitary crisis on French healthcare workers: Risk factors and coping strategies. Front. Psychiatry.

[14] Kamberi F., Sinaj E., Jaho J., Subashi B., Sinanaj G., Jaupaj K., Stramarko Y., Arapi P., Dine L., Gurguri A. (2021). Impact of COVID-19 pandemic on mental health, risk perception and coping strategies among health care workers in Albania—evidence that needs attention. Clin. Epidemiol. Glob. Health.

[15] World Health Organization (2022). Timeline: WHO’s COVID-19 Response.

[16] Braun V., Clarke V. (2006). Using thematic analysis in psychology. Qual. Res. Psychol..

[17] Braun V., Clarke V. (2013). Successful Qualitative Research: A Practical Guide for Beginners.

[18] Clarke V., Braun V. (2017). Thematic analysis. J. Posit. Psychol..

[19] VERBI Software (2019). MAXQDA 2020 [Computer Software].

[20] Lincoln Y.S., Guba E.G. (1985). Naturalistic Inquiry.

[21] Guba E., Lincoln Y., Denzin N., Lincoln Y. (1994). Competing paradigms in qualitative research. Handbook of Qualitative Research.

[22] Schug C., Morawa E., Geiser F., Hiebel N., Beschoner P., Jerg-Bretzke L., Albus C., Weidner K., Steudte-Schmiedgen S., Borho A. (2021). Social support and optimism as protective factors for mental health among 7765 healthcare workers in Germany during the COVID-19 pandemic: Results of the VOICE Study. Int. J. Environ. Res. Public Health.

[23] Smith A.J., Shoji K., Griffin B.J., Sippel L.M., Dworkin E.R., Wright H.M., Morrow E., Locke A., Love T.M., Harris J.I. (2022). Social cognitive mechanisms in healthcare worker resilience across time during the pandemic. Soc. Psychiatry Psychiatr. Epidemiol..

[24] Reilly S.E., Soulliard Z.A., McCuddy W.T., Mahoney J.J. (2021). Frequency and perceived effectiveness of mental health providers’ coping strategies during COVID-19. Curr. Psychol..

[25] Franck E., Haegdorens F., Goossens E., Gils Y.V., Portzky M., Somville F., Abuawad M., Slootmans S., Bogaert P.V. (2021). The role of coping behavior in healthcare workers’ distress and somatization during the COVID-19 pandemic. Front. Psychol..

[26] Argyroula K., Alexandra T., George T. (2022). From secondary traumatic stress to vicarious posttraumatic growth amid COVID-19 lockdown in Greece: The role of health care workers’ coping strategies. Psychol. Trauma: Theory Res. Pract. Policy.

[27] Patel H., Easterbrook B., D’Alessandro-Lowe A., Andrews K., Ritchie K., Hosseiny F., Rodrigues S., Malain A., O’Connor C., Schielke H. Associations between trauma and substance use among healthcare workers and public safety personnel during the SARS-CoV-2 (COVID-19) pandemic: The mediating roles of dissociation and emotion dysregulation. Eur. J. Psychotraumatol..

[28] Phoenix Australia Centre for Posttraumatic Mental Health, Candian Centre of Excellence—PTSD (2020). Moral Stress Amongst Healthcare Workers during COVID-19: Moral Injury Guide.

[29] Folkman S. (2010). Stress, coping, and hope. Psycho-Oncology.

[30] Frydenberg E. (2004). Coping competencies: What to teach and when. Theory Into Pract..

